# Comparative Proteomic Analysis of the Venoms from the Most Dangerous Scorpions in Morocco: *Androctonus mauritanicus* and *Buthus occitanus*

**DOI:** 10.3390/life13051133

**Published:** 2023-05-05

**Authors:** Ines Hilal, Soukaina Khourcha, Amal Safi, Abdelaziz Hmyene, Syafiq Asnawi, Iekhsan Othman, Reto Stöcklin, Naoual Oukkache

**Affiliations:** 1 Laboratory of Venoms and Toxins, Pasteur Institute of Morocco, Casablanca 20360, Morocco; 2 Laboratory of Biochemistry, Environment and Food Technology, Faculty of Sciences and Techniques of Mohammedia, Mohammedia 20650, Morocco; 3 Jeffrey Cheah School of Medicine and Health Sciences, Monash University Malaysia, Bandar Sunway 47500, Malaysia; 4 Atheris Laboratories, Case Postale 314, CH-1233 Bernex, Geneva, Switzerland

**Keywords:** *Androctonus mauritanicus*, Buthus occitanus, mass spectrometry, mass fingerprinting, proteomics, scorpion, venom

## Abstract

Morocco is known to harbor two of the world’s most dangerous scorpion species: the black *Androctonus mauritanicus* (*Am*) and the yellow *Buthus occitanus* (*Bo*), responsible for 83% and 14% of severe envenomation cases, respectively. Scorpion venom is a mixture of biological molecules of variable structures and activities, most of which are proteins of low molecular weights referred to as toxins. In addition to toxins, scorpion venoms also contain biogenic amines, polyamines, and enzymes. With the aim of investigating the composition of the *Am* and *Bo* venoms, we conducted an analysis of the venoms by mass spectrometry (ESI-MS) after separation by reversed-phase HPLC chromatography. Results from a total of 19 fractions obtained for the *Am* venom versus 22 fractions for the *Bo* venom allowed the identification of approximately 410 and 252 molecular masses, respectively. In both venoms, the most abundant toxins were found to range between 2–5 kDa and 6–8 kDa. This proteomic analysis not only allowed the drawing of an extensive mass fingerprint of the *Androctonus mauritanicus* and *Buthus occitanus* venoms but also provided a better insight into the nature of their toxins.

## 1. Introduction

From their iconic appearance to their venomous stings, scorpions are a captivating group of arthropods. These living fossils have persisted and kept their morphological characteristics for more than 400 million years [1]. They are represented by over 2000 different species classified into six families: *Bothriuridae, Scorpionidae, Buthidae, Vejovidae, Chlaerilidae,* and *Chactidae* [2,3]. The *Buthidae* family is widespread around the world, with 86 genera and 990 species. Scorpions belonging to this family are the most widely distributed and include the most dangerous genera, the *Androctonus* and *Buthus* in North Africa, *Parabuthus* in South Africa, *Leiurus* in the Near and Middle East, *Tityus* in South America, *Centruroides* in North and Central America and *Mesobuthus* in Asia [4,5,6,7,8]. Like most venomous animals, scorpions use their venom for predation or defense when threatened. This viscous secretion has a complex and specific composition; it consists of a cocktail of substances such as biogenic amines (histamine and serotonin), polyamines, and many enzymes [9,10]. However, the majority of scorpion venom bioactive molecules are peptides and small proteins. These toxins are mostly active on the ionic channels Na^+^ [11,12], K^+^ [13,14,15], Ca^2+^ [16], and Cl^−^ [17], on which they act with high potency and selectivity. These neurotoxins are characterized by a low molecular weight and a huge diversity of structures and modes of action that disrupt the transmission of nerve impulses [18]. Scorpion envenomation is a life-threatening emergency and a critical public health issue [19,20], causing more than 1.5 million cases, of which 2600 are lethal, especially in children [21]. This number is worryingly still growing over the world each year, and many cases remain unreported. In Morocco, scorpion envenomation is the leading cause of intoxication, accounting for 30 to 50% of cases reported by the Moroccan Poison Control Center, with 25,000 to 40,000 stings annually [22]. Most of the stings are observed in the southwestern provinces of the kingdom, in Kalaat-sraghna, El-Jadida, Agadir, and Tan-Tan [23], where the most incriminated species are mainly *Androctonus mauritanicus* (*Am*) known as ‘the black scorpion’ followed by *Buthus occitanus* (*Bo*) ‘the yellow scorpion’ [24,25,26]. During a sting, the toxins of these venoms diffuse rapidly from the injection site to different vascular compartments and induce peripheral nervous system stimulation with a massive release of neurotransmitters and cell mediators, thus generating various pathophysiological disorders at all the organic systems [27]. An exhaustive screening of scorpion venom bioactive molecules will be a good source of novel pharmacological tools for studying the toxins and understanding their activities, improving envenomation therapeutics, and discovering new drug candidates [28]. Until recently, different analytical techniques were used for the characterization of scorpion venom; most of the current knowledge has been obtained by conventional biochemical and pharmacological approaches, which consist in targeting the toxins or the fractions of interest without making an exhaustive characterization of the total venom of the scorpion, thus minimizing, indirectly, the characterization of other toxins or other venom components [29,30].

A new era in the characterization of scorpion venoms was developed, the venomics strategies allowing a major knowledge on the biochemical constitution of venoms of high potential impact in medicine and beyond. Mass spectrometry and next-generation sequencing remain the most widely used and well-documented [31]. Different types of mass spectrometers give access to a large amount of knowledge, going from simple molecular masses of intact components to primary sequences of peptides [32,33]. The most widely used is ESI-MS because its advantage of being more accurate and sensitive in mass determination [34]. Even though several studies have been done for these two venoms [25,35,36,37,38,39], no comparative proteomic study has been conducted before. The present work offers an exhaustive view of the mass fingerprinting of the most dangerous scorpions in Morocco, *Androctonus mauritanicus* and *Buthus occitanus*, using the proteomic strategies focusing on mass spectrometry, intending to obtain more fundamental knowledge on the compositional, toxical, and structural characteristics of these venoms.

## 2. Methods

### 2.1. Ethical Statements

The animals were handled according to the ethical guidelines adopted by the World Health Organization (WHO) and approved by a local Ethics Committee of the Institut Pasteur of Morocco under agreement number 8.3.A-2015.

### 2.2. Venom Preparation

A total of 500 specimens (male and female, juvenile and adult) of *Androctonus mauritanicus* and *Buthus occitanus* were captured from the region of Essaouira, where scorpion envenomation cases are recorded in abundance. The scorpions were kept in captivity in the scorpionarium of the Pasteur Institute of Morocco, with water ad libitum, and fed with insects in individual boxes to avoid scorpion cannibalism. The crude venom was milked by electrical stimulation, pooled, centrifuged at 12,000× *g* for 20 min, frozen, freeze-dried, and kept at −20 °C until use [26]. The concentration of the protein content of the venoms was determined using the estimation at 280 nm method, assuming that 1 unit of absorbance in a quartz cuvette with 1 cm optical path equals 1 mg/mL protein concentration [40,41].

### 2.3. Venom Lethality (LD_50_)

Lethal potency of the venoms was evaluated by measuring the Median Lethal Dose 50 (LD_50_) following the recommendations of the World Health Organization (WHO) [42]. The LD_50_ represents the dose that kills 50% of a homogeneous population of Swiss mice (18–22 g). Increasing doses of *Am* and *Bo* venoms were adjusted in isotonic NaCl solution, then injected, and mortality rates were recorded after 24 h. Two injection routes were used: intravenous (IV) and intraperitoneal (IP) [43]. The analysis of the different results was done using the GraphPad Prism 5 software (Version 5, Dotmatics, Boston, MA, USA) in accordance with the supplied algorithm [44]. 

### 2.4. Venom Separation by SDS-PAGE

Following Laemmli SDS-PAGE method [45], electrophoretic analysis of *Am* and *Bo* venoms was performed on 15% polyacrylamide gel under reducing conditions in the presence of SDS. All samples were dissolved in a sample buffer (50 mM Tris–HCl, pH 6.8, 0.1 M DTT, 10% glycerol, 2% SDS, and 0.1% bromophenol blue). A constant electric current of 70 mA was applied for two hours. After migration, the gel was stained with Coomassie Brilliant Blue R250 [46,47]. Molecular weights were estimated using standard low-rank markers (Bio-Rad, Hercules, CA, USA).

### 2.5. Venom Fractionation by RP-HPLC

Crude venom samples were resuspended at 1 mg/mL 0.1% TFA in water (solution A) and submitted to a solid phase extraction on a Sep-Pak Plus C18 cartridge (360 mg, 55–105 μm, Waters, Milford, MA, USA), conditioned with methanol and equilibrated with solution A. After loading and washing with 10 mL solution A, the elution of peptides and proteins was performed using 3–5 mL of solution B (0.1% TFA in 70% acetonitrile and 30% water). The eluate was collected and freeze-dried on a SpeedVac concentrator (SC 250 DDA SpeedVac Plus, Thermo Savant, Waltham, MA, USA).

*Am* and *Bo* extracts (1 mg) were subsequently fractionated by RP-HPLC using a C-18 analytical column (4.6 × 250 mm, 4 µm particle size, 300 A pore size) as previously described [48]. Briefly, the column was equilibrated with solvent A, and fractions were eluted using a 0–100% gradient of solvent B (acetonitrile/0.08% TFA) over 120 min at a flow rate of 1.0 mL/min at 25 °C. The elutate medium was monitored by UV absorbance at wavelength of 280 nm, and fractions were collected using an automated Gilson fraction collector at detector output. Three different HPLC runs were performed.

### 2.6. Mass Spectrometry Analysis

An aliquot of each fraction obtained by RP-HPLC has been submitted to a mass spectrometry (online LC-ESI-MS) analysis. Peptide profiles were assessed using an Alliance 2795 HPLC separation module (Waters, Milford, MA, USA) fitted with a post-column split; 5% of the eluate was directed towards the electrospray ionization source of a Quattro Micro mass spectrometer (Micromass-Waters, Milford, MA, USA) and 95% towards a 2487 UV diode array detector (Waters), using a 1%/min gradient of acetonirine in 0.1% formic acid (FA) in water. The Masslynx 4 Micromass^®^ software (Waters, Milford, MA, USA) was used for data analysis.

### 2.7. Tryptic Digestion

The fractions obtained by HPLC (0.5 mg) were mixed with 25 μL of 100 mM ammonium bicarbonate (pH 7.0), 25 μL of trifluoroethanol and 1 μL of 200 mM DTT, agitated and incubated at 90 °C for 20 min. After cooling samples at room temperature, proteins were alkylated with 4 μL of 200 mM iodoacetamide in the dark at room temperature for 1 h. Excess of iodoacetamide was blocked by addition of 1 μL DTT through incubation for 1 h at room temperature. Samples were diluted with water and ammonium bicarbonate to adjust pH (7–9). Proteolytic digestion was performed using a trypsin solution at a ratio of 1/20 (enzyme/substrate), followed by overnight incubation at 37 °C. Trypsin activity was removed using 1 μL FA. The samples were freeze-dried and stored at −20 °C until use.

### 2.8. LC/MS/MS Characterization

Online LC/MS/MS of venom samples dissolved in 0.1% TFA to a concentration of 1 mg/mL was performed using a C8 analytical column (75 µm × 43 mm, 5 µm particle size, 300 Å) with solvent A (0.1% TFA) and solvent C (90% acetonitrile in 0.1% TFA). Electrospray mass spectra were acquired on a PE-SCIEX API 300 LC/MS/<MS system with an Ionspray atmospheric pressure ionization source. Samples (1 µL) were infused into the LC/MS/MS system and analyzed in positive ionization mode. Full scan data were acquired at an orifice potential of 80 V over the ion range 600–3000 *m*/*z* with a step size of 0.2 u. Data processing was performed with the aid of the software package Biomultiview (PE-SCIEX, Concord, ON, Canada). MS/MS analysis and N-terminal sequence were straightforwardly assigned by BLAST analysis (http://www.ncbi.nlm.nih.gov/BLAST) to a previously reported protein or to a known protein family.

## 3. Results

### 3.1. Protein Quantification

The results obtained revealed that the yield of proteins after the SepPak extraction was 0.897 and 0.771 mg/mL for the *Am* and *Bo* venoms, respectively.

### 3.2. Lethality of A. mauritanicus and B. occitanus Venoms 

The toxicity of the venoms was determined by measuring the LD_50_ with 95% confidence intervals. The results show that the *Am* venom is three times more toxic compared to that of the *Bo* (Table 1). The LD_50_ measured using the intravenous route (IV) was almost the same as that obtained by the intraperitoneal route (IP). This reveals that the molecules responsible for mortality in the case of scorpion envenomation are molecules with low molecular weights diffusing rapidly through the bloodstream and that their bioavailability is very high. 

### 3.3. Electrophoretic Profile

**Figure 1:**
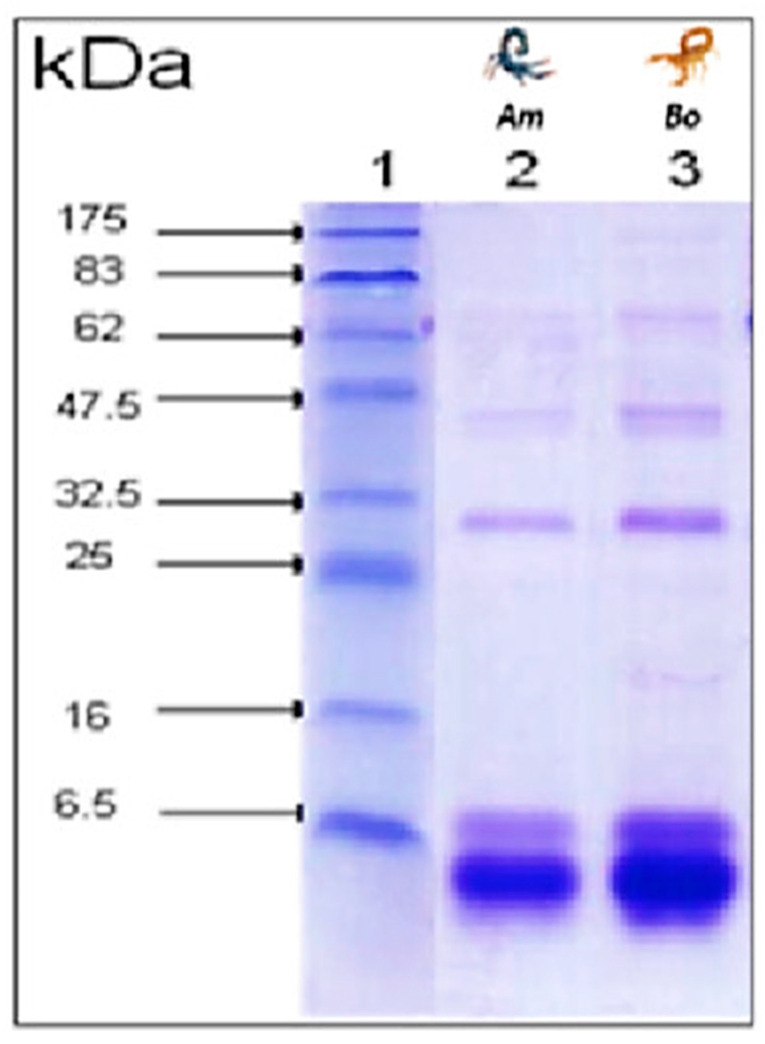
Electrophoretic profile of venoms on polyacrylamide gel in the presence of SDS in reducing conditions. Lane 1: molecular mass markers, Lane 2: *Am* venom, Lane 3: *Bo* venom.

The venom proteins were analyzed by SDS-PAGE separation and Coomassie staining. Electrophoresis analysis revealed that the venom consisted primarily of one major protein band with a molecular weight of approximately 6.5 kDa, consistent with low molecular weight toxins (Figure 1).

### 3.4. HPLC 

**Figure 2:**
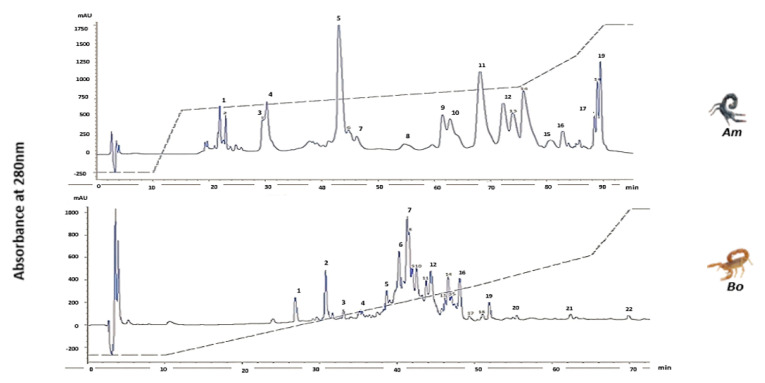
Reversed-phase HPLC profile of 1 mg protein of *Am* and *Bo* venoms performed with a linear gradient from solvent A (0.1% TFA in water) to 100% solvent B (0.10% TFA in acetonitrile) at a flow rate of 1 mL/min over 120 min.

The HPLC chromatograms revealed the complexity of the venoms, which were found to contain hundreds of bioactive molecules with diverse biological properties. Interestingly, each venom displayed a unique profile, underscoring the distinctiveness of each species. HPLC analysis facilitated the isolation of 19 fractions from the *Am* venom and 22 from the *Bo* venom (Figure 2), suggesting that the *Bo* venom is more complex than that of the *Am*.

### 3.5. Mass Fingerprinting of Am and Bo Venoms

**Figure 3:**
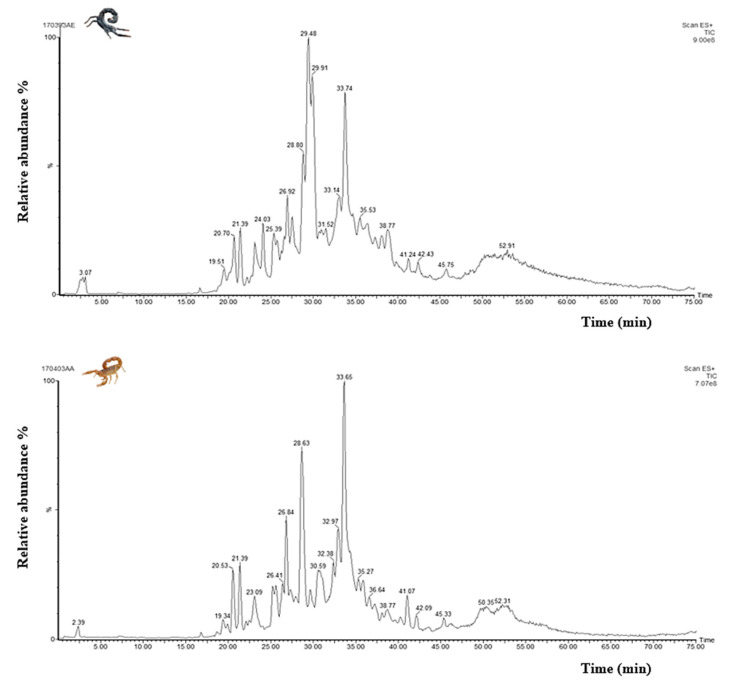
Total ion current chromatograms generated by LC-MS analysis of *Am* and *Bo* venoms.

Through the analysis of the different fractions obtained from HPLC separation, we were able to detect 410 and 252 different molecular masses in the *Am* and *Bo* venoms, respectively (Figure 3). These findings confirmed that the *Am* venom is richer in molecules than the *Bo* venom, potentially explaining why it is associated with more severe cases of envenomation.

**Figure 4:**
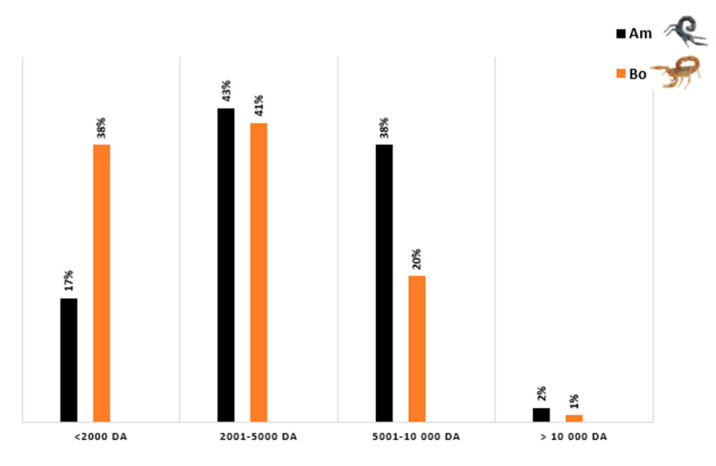
Molecular mass distribution of *Am* and *Bo* Venoms.

Our initial proteomic approach allowed us to create a mass fingerprint of each venom by identifying molecular masses between 500 and 8000 Da for the *Am* venom and 500 and 7000 Da for the *Bo* venom, which were then categorized into different ranges. In both venoms, the greatest number of signals were identified in masses ranging from 2–5 kDa, followed by those over 5 kDa in the case of the *Am* venom and under 2 kDa for the *Bo* venom. Although other masses were also identified, they were present at a lower percentage, particularly those over 10 kDa (Figure 4).

### 3.6. Composition of Am and Bo Venoms

Based on the results of our comparative analysis, we found that the *Am* venom is composed of 79% neurotoxins (relative values based on MS signal intensities), with 47% of these toxins targeting Na^+^ channels, referred to as NaScTxs. Among NaScTxs, α-NaScTxs were found to be more abundant than β-NaScTxs, making up 88% of the total. We also identified Toxin AaHIT4 (P21150), which can target both site 3 and site 4 of the sodium channel. Additionally, we detected α and β-KScTxs, which account for 23% of the total composition. Toxins targeting Cl^−^ channels (ClScTxs) and Ca^2+^ channels (CaScTxs) were less prevalent, constituting only 6% and 3%, respectively. The enzymatic composition was estimated at 12%.

**Figure 5:**
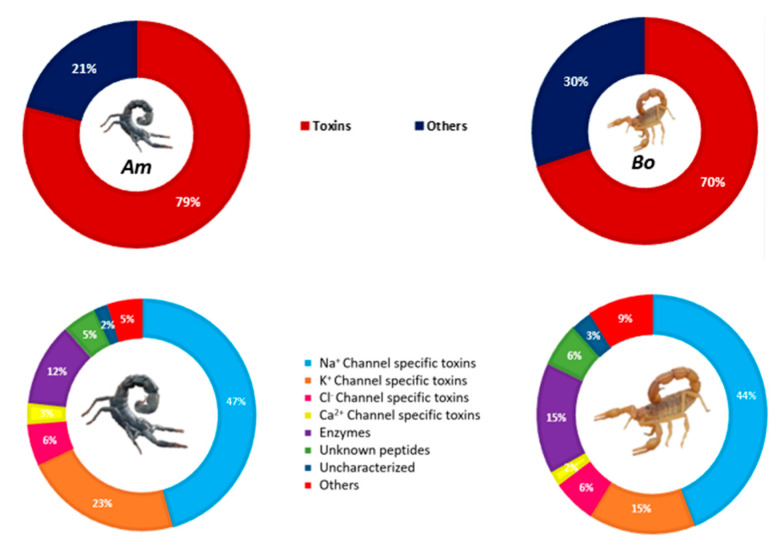
*Am* and *Bo* venoms components.

Concerning the *Bo* venom, neurotoxins were once again the major toxins, representing 70% of the composition, with 44% targeting Na^+^ channels. Both α-NaScTxs and β-NaScTxs were detected, with a predominance of the alpha group comprising 75% of NaScTxs. KscTxs, ClScTxs, and CaScTxs represented 15%, 6%, and 2% of the total composition, respectively, while enzymes were estimated to make up 15% (Figure 5).

**Figure 6:**
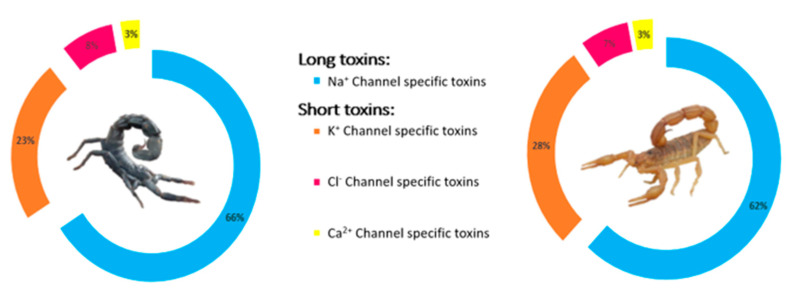
Percentage of toxins in *Am* and *Bo* venoms.

The toxic fractions of the venoms of both *Am* and *Bo* contain a high proportion of NaScTxs, which constitute more than 60% of the total venom content (Figure 6). These long toxins, with molecular masses ranging from 4 to 8 kDa, exert their biological effects on both the peripheral and central nervous systems. Remarkably, NaScTxs are the main contributors to the mortality caused by these venoms, accounting for over 73% of the lethal effects. In contrast, the shorter toxins found in the 3–4 kDa mass range, such as KScTxs, ClScTxs, and CaScTxs, predominantly affect the function of these specific ion channels. These toxins display activity only on the central nervous system and contribute to the toxic effects of the venom but not to its lethality, as is the case for the NaScTxs.

### 3.7. Mass Spectrometry Identification

MS/MS data processing was performed using the ProteomeDiscover 2.2 software (Thermo Fisher Scientific, Waltham, MA, USA), and the identification of the different peptides/proteins was achieved by sequence homology, querying the Uniprot database (https://www.uniprot.org). The identified proteins were classified into different families according to their function by referring to the UniProt and InterPro databases (https://www.ebi.ac.uk). The list of potential proteins obtained from the analysis has been inserted in a dedicated of proteins and related peptides identifiable with sequences matching known proteins. The analysis of the mass spectrometry data also indicated that there is a high sequence homology with other scorpion venoms species such as *Leiurus quinquestriatus quinquestriatus, Androctonus australis, Mesobuthus martensii,* and *Lychas mucronatus.*

Previously identified peptides were found in the *Am* venom, corresponding to neurotoxins, namely alpha-toxin Amm3, alpha-toxin Amm5, alpha-toxin Amm8, neurotoxin P2, potassium channel toxin alpha-KTx 15. 3, potassium channel toxin alpha-KTx 3.1, potassium channel toxin alpha-KTx 5.2 and potassium channel toxin alpha-KTx 8.1 (Table 2).

However, some identified sequences share sequence similarities with peptides characterized in the venom of other scorpions. Thus, for the *Am* venom, we found 19 homologies of sequences matching other scorpion venom peptides (Table 2).

Similarly, the *Bo* venom also contained peptides corresponding to previously identified neurotoxins, including alpha-like toxin Bom3, alpha-like toxin Bom4, alpha-mammal toxin Bot3, alpha-toxin Bot1, alpha-toxin Bot11, Beta-toxin BotIT2, and Neuro-toxin Bot2. Interestingly, a peptide previously identified in the venom of the viper *Daboia russelli siamensis* was also found in the *Bo* venom (Table 3). In total, 71% of the peptides in the *Bo* venom showed similarity with other species, while the *Am* venom shared 61% of its peptides with other species. This finding is of great importance in the development of antivenoms with a broad spectrum of protection.

## 4. Discussion

Despite their small size, Scorpions are feared for their potent venom. It is a complex mixture of different components, of which folded peptides are the most dominant [49,50]. They have been studied for decades using conventional bioactivity-guided approaches broadly used with natural substances that consist of purifying the biomolecule of interest prior to studying its structure and function. Unfortunately, these studies only reflected a partial picture of the whole venom, and the information obtained is in favor of the abundant toxins in the venoms of the most incriminated species, leaving aside those rare or more difficult to collect that remain largely unexplored. Analytical studies of venoms have ongoingly improved with the help of technological developments. The implementation of recent venomics approaches (mass spectrometry, NextGen sequencing) in the field facilitated the obtaining of information from these matrices [51].

Venom profiling by mass spectrometry initiated in the early Nineties remains a fundamental approach to global venom exploration. Such data, with or without chromatographic fractionation, produces a global picture of the venom and reveals its complex composition. For this purpose, a mass fingerprint of the Moroccan scorpion venoms of *Androctonus mauritanicus* and *Buthus occitanus* was performed after an HPLC separation and by using mass spectrometry (ESI-MS and ESI-LC/MS/MS).

LD_50_ results have confirmed what was already known: *A. mauritanicus* and *B. occitanus* venoms are very toxic. Previous biochemical characterization studies had reported the medical importance of *A. mauritanicus* and *B. occitanus,* which are involved in 83% and 14% of envenomation cases in Morocco, respectively. These studies have shown that *A. mauritanicus* is the most dangerous scorpion in Morocco [52]. Its venom is highly toxic, with an LD_50_ of 2.4 µg/mouse, and responsible for adverse pathophysiological effects and intense electrolyte imbalance. Meanwhile, *B. occitanus* is considered the second most dangerous scorpion in the kingdom, with an LD_50_ of 5.7 µg/mouse [36,38,43].

Buthidae venoms are known to be harmful since numerous of their components (especially NaTxs) have an affinity for human receptors [53]. The main difference between the venom of a Buthidae and a non-Buthidae scorpion is that NaTxs are predominant and more abundant in the venom of the Buthidae family [17,54,55,56]. They are responsible for the lethality, the neurotoxic effects and have a leading role in the complications of scorpionism [57]. According to their physiological effects on voltage-gated sodium ion channels, NaTxs can be divided into two groups, named α-NaTx and β-NaTx [58]. The difference between these two groups is that α-NaTxs bind to site 3 and delay or inhibit the channel’s normal inactivation process, while β-NaTxs bind to site 4 and encourage the channel opening at more negative membrane potentials [59].

This study correlates well with previous works of proteomic analysis of scorpion venom, in which toxins that impair Na^+^ and K^+^ channels constitute the main toxin components of Buthidae venoms [60,61]. Thus, our findings show that the majority of mass in *A. mauritanicus* and *B. occitanus* venoms are composed of long toxins that target Na^+^ channels, accounting for 66% and 62%, respectively. Short toxins, which act on K^+^, Cl^−^, and Ca^2+^ ion channels, constitute 34% of *A. mauritanicus* venom and 38% of *B. occitanus* venom. On the other hand, peptides with molecular masses less than 2 kDa and enzymes with molecular masses greater than 10 kDa are less abundant in both venoms. These results corroborate previous studies on *A. mauritanicus* and *B. occitanus* venoms, which identified NaScTxs and KScTx neurotoxins as the main components [25,39,62,63,64,65,66,67,68,69,70,71].

Similarly, the proteomic analysis of the venom of the species *Centruroides tecomanus* (family Buthidae) showed an abundance of molecular masses (7–8 kDa) corresponding to neurotoxins targeting voltage-gated Na^+^ channels followed by those corresponding to neurotoxins acting on K^+^, Cl^−^, and Ca^2+^ ion channels (4–5 kDa) [72]. A neurotoxin-rich composition has been demonstrated as well in other venoms of scorpions belonging to the Buthidae family: *Tityus serrulatus, Centruroides limpidus, Centruroides hirsutipalpus, Mesobuthus martensii, Tityus metuendus, Androctonus bicolor* and *Mesobuthus tamulus* as well as to the Scorpionidae family: *Heterometrus longimanus* and *Heterometrus petersii* [49,72,73,74,75,76,77]. At the same time, the venom composition of the scorpions *Rhopalurus agamemnon* (family Buthidae), *Megacormus gertschi* (family Euscorpiidae), and *Thorellius atrox* (family Vaejovidae) present a composition rich in enzymes [78,79,80].

The abundance of toxins targeting Na^+^ channels in these venoms explains why these scorpions are so dangerous, as these toxins are responsible for mortality. This raises the hypothesis that scorpions can be classified according to their dangerousness based on the percentage of sodium-channel toxins present in their venom. Noteworthy, the development of an adjuvant able to block Na^+^ channel receptors would significantly reduce the mortality rate in populations at high risk of scorpionism. In contrast, short toxins specific to K^+^, Cl^−^, and Ca^2+^ channels are responsible for toxicity and are less involved in lethality.

A total of 410 masses were found in the *Am* venom and 252 masses in that of the *Bo.* Although more molecular weights have been detected in the venom of *Leiurus quinquestriatus hebraeus* (554 masses), *Tityus stigmurus* (632 masses), and *Pandinus cavimanus* (700 masses), fewer compounds were obtained from the venoms of *Centruroides limpidus* (52 masses), *Androctonus crassicauda* (80 masses), *Tityus costatus* (90 masses), *Tityus pachyrus* (104 masses), *Mesobuthus tamulus* (110 masses), *Tityus serrulatus* (147 masses), *Rhophalurus junceus, Tityus metuendus* and *Mesobuthus martenssi* (200 amasses), *Serradigitus gertschi* (204 masses), *Tityus discrepans* (205 masses), *Paravaejovis schwenkmeyeri* (212 masses), and *Rhopalurus agamemnon* (230 masses) [72,75,77,80,81,82,83,84,85,86,87,88,89,90,91,92,93].

However, we noted that *A. mauritanicus* scorpion venom is richer in NaScTxs that target mammalian Na^+^ channels, namely the alpha toxin Amm5, which is considered the most lethal toxin identified so far in Moroccan scorpion venom [25]. In addition, the identification of alpha-toxin Amm3 and the alpha-toxin-like toxin Lqq 5, the most lethal toxin of the scorpion Leiurus quinquestriatus that shares a 95.3% sequence similarity with alpha-toxin Amm 5, explains why the venom of the scorpion *A. mauritanicus* is estimated to be the most toxic and responsible for the most severe envenomations in Morocco [52,70].

These results illustrate the great polymorphism of the toxins of scorpions *A. mauritanicus* and *B. occitanus*. Among those involved in the pathophysiology of envenomations, we found NaScTxs and KscTxs; these two families work in synergy to generate a prolonged depolarization of the cell membrane and thus a neuronal excitation which causes the stimulation of the sympathetic and parasympathetic nervous system leading to the release of cellular mediators responsible for all the alterations observed during a scorpion envenomation. The high content of these neurotoxins in the venoms *A. mauritanicus* and *B. occitanus* explains their toxicity and their involvement in the most serious cases of envenomation in our country.

## 5. Conclusions

Herein, we have demonstrated that a multi-faceted proteomic strategy, including cutting-edge separation and characterization techniques, can provide valuable insights into the composition and toxicology of scorpion venoms. Specifically, our results show that the venoms of *Androctonus mauritanicus* and *Buthus occitanus* scorpions contain a highly complex mixture of hundreds of distinct peptides, mainly neurotoxins, with NaScTxs and KScTxs as the predominant components, representing 70% and 59% of the venom composition, respectively. By elucidating the toxicological profiles of these venoms, our findings provide a critical foundation for improving the understanding of the pharmacological mechanisms involved in envenomation and for developing effective antivenom therapies. Overall, this study highlights the importance of using advanced proteomic techniques for the characterization and analysis of complex biological samples, with broad implications for biomedical research and drug discovery.

## Figures and Tables

**Table 1:** Comparison of median lethal dose (LD_50_) of *Am* and *Bo* venoms.

Venoms	LD_50_	
	*Am* Venom	*Bo* Venom
IV (µg/mouse)	4.7 (4.1–5.4)	15.2 (14.8–15.6)
IP (µg/mouse)	5.8 (5.3–6.4)	17.1 (16.7–17.5)

**Table 2:** Summary of the proteins associated with *Am* from the fractions obtained from the reverse phase HPLC fractionation and further analysis on LC/MS/MS.

Proteins Associated with *Am*					
Sample/Fraction	Protein Name	Molecular Weight (Da)	Sequence	Swiss-Prot ID	Coverage (%)
A5, A6, A7, A8, A9, A10	Alpha-toxin Amm3 OS = Androctonus mauritanicus mauritanicus	7009.1	(K)PVINITWLR NSKSVTDG(L)	P0C910	92.9
A7, A8, A9, A10, A11	Alpha-toxin Amm5 OS = Androctonus mauritanicus mauritanicus	7301.3	(−)LKDGYIIDDLNCTFFCGR(N)	P01482	89.7
A5, A6, A7	Alpha-toxin Amm8 OS = Androctonus mauritanicus mauritanicus	9654.2	(K) LPDHVR (T)	Q7YXD3	94.4
A1, A4, A5, A6	Neurotoxin P2 OS = Androctonus mauritanicus mauritanicus	3673.3	(−) CGPCFTTDPYTESK (C)	P01498	93.8
A4, A5	Potassium channel toxin alpha-KTx 15.3 OS = Androctonus mauritanicus mauritanicus	3845.6	(K) VIGVAAGK (C)	P60208	96.3
A2, A3	Potassium channel toxin alpha-KTx 3.1 OS = Androctonus mauritanicus mauritanicus	4156	(K) CSGSPQCLKPCK (D)	P24662	88
A3, A4	Potassium channel toxin alpha-KTx 5.2 OS = Androctonus mauritanicus mauritanicus	3421.2	(R) SLGLLGKCIGVK (C)	P31719	95.8
A2, A3, A4, A5, A7, A8, A11	Potassium channel toxin alpha-KTx 8.1 OS = Androctonus mauritanicus mauritanicus	3184.6	(−) VSCEDCPEHCTQK (A)	P56215	94
**Proteins Associated with other Species**					
**Sample/Fraction**	**Protein Name**	**Molecular** **Weight (Da)**	**Sequence**	**Swiss-Prot ID**	**Coverage** **(%)**
A9, A10, A16, A17	Alpha-mammal toxin Lqq5 OS = Leirus quinquestriatus quinquestriatus	7301.3	(−)LKDGYIVDDKNCTFFCGR(N)	A7NGC5	77.3
A8	Alpha-toxin BeM10 OS = Buthus eupeus	7373.4	(R) NAYCDEECK (K)	P01490	70.6
A6, A14	Beta-bungarotoxin BF B1 chain OS = Bungarus fasciatus	9636.2	(R) AFYYLPSAK (R)	B2KTG2	71
A8	Beta-insect excitatory toxin 1 OS = Androctonus australis	9852	(K)KNGYAVDSSG(K)	P01497	99.5
A7	Beta-insect excitatory toxin 2 OS = Androctonus australis	9862.7	(R)YAVDSSGK APECLLSN(K)	P15147	82.5
A8	Beta-insect excitatory toxin LqhTTlc OS = Leirus quinquestriatus hebraeus	9935.8	(K) VMEISDTR (K)	P68723	77.3
A6, A7, A8, A9, A10	Beta-toxin KAaM1 OS = Androctonus australis	9104.8	(K) YGYCYAFQCWCEYLEDK (N)	Q4LCT0	97.3
A6, A7, A12, A19	Neurotoxin BmK-M10 OS = Mesobuthus martensii	9346.0	(K) YGNACWCIK (L)	Q61705	85.4
A13, A15, A16	Toxin Aah4 OS = Androctonus australis	9156.7	(K) NCVYHCYPPCGLCK (K)	P45658	82.3
A17, A18	Neurotoxin LmNaTx34.5 (Fragment) OS = Lychas mucronatus	9472.2	(K)GGSYGYCYFWK(L)	POC16	73.1
A18	Potassium channel toxin AaTXK-beta OS = Androctonus australis	10,148.2	(R)TILQTVVHK(V)	P69939	87.6
A10, A11	Neurotoxin LmNaTx34.5 (Fragment) OS = Lychas mucronatus	9472.2	(R)AGREKGCK VWCVIN(N)	P0CI60	88.2
A4, A5	Neurotoxin-1 OS = Androctonus australis	9061	(C)VYHCVPPCDGLCK(K)	P01479	99.6
A7, A8, A9	Neurotoxin-like protein STR1 OS = Androctonus australis	7640.8	(R) DGYIVHDGTNCK (Y)	P80950	83.8
A11	Potassium channel toxin AaTXK-beta OS = Androctonus australis	10,148.2	(R)TILQTVVHK(V)	P69939	87.6
A2	Potassium channel toxin alpha-KTx 3.4 OS = Leirus quinquestriatus hebraeus	4020.9	(K) CTGSPQCLKPCK (D)	P46110	85.5
A2, A3	Potassium channel toxin alpha-KTx 3.9 OS = Buthus occitanus tunetanus	4028	(C)KDAGMRFGKCMNRK(C)	P59290	98.7
A5, A6, A7, A8, A9	Potassium channel toxin BmTXK-beta OS = Mesobuthus martensii	10,430.6	(K) LTSMSEYACPVIEK (W)	Q9NJC6	85.6
A4, A5, A6, A7	Potassium channel toxin BmTXK-beta-2 OS = Mesobuthus martensii	10,212.2	(K) TQFGCPAYQGYCDDHCQDIK (K)	Q9N661	78.4
A8, A9, A10, A11	Toxin AaHIT4 OS = Androctonus australis	7786.0	(R) KSELWNYK (T)	P21150	97
A4	Toxin BmKaITI OS = Mesobuthus martensii	9649.3	(R) DAYIAQNYNCVYCAR (D)	Q9GQW3	88.2
A9	Toxin BmTxKS4 OS = Mesobuthus martensii	8856	(K) GHSSCTNGLEMTEEDF (C)	Q5F1N4	99.7

**Table 3:** Summary of the proteins associated with *Bo* from the fractions obtained from the reverse phase HPLC fractionation and further analysis on LC/MS/MS.

Proteins Associated with *Bo*					
Sample/Fraction	Protein Name	MW	Sequence	Swiss-Prot ID	% Coverage
B8, B9, B10	Alpha-like toxin Bom3 OS = Buthus occitanus mardochei	6871.8	(K)LPDKVPIKVPGK(C)	P13488	93.4
B8, B12, B13	Alpha-like toxin Bom4 OS = Buthus occitanus mardochei	7296.3	(K)YGNACWCEDLPDNVPIRIPGK(C)	P59354	91.3
B8	Alpha-mammal toxin Bot3 (Fragment) OS = Buthus occitanus tunetanus	8059.1	(K)LKGESGYCQWASPYGNACYCYKLPDHVR(T)	P01485	83.3
B8, B9, B10, B11	Alpha-toxin Bot1 OS = Buthus occitanus tunetanus	7268.2	(K)DLPDNVPIRIPGK(C)	P01488	88.2
B8	Alpha-toxin Bot11 OS = Buthus occitanus tunetanus	7468.5	(R)YGNACWCYKLPDHVR(T)	P01486	92.0
B11, B12	Beta-toxin BotIT2 OS = Buthus occitanus tunetanus	6917.8	(K)WGLACWCEDLPDEK(R)	P59863	82.1
B13	Lipolysis-activating peptide 1-beta chain OS = Buthus occitanus tunetanus	10,386.3	(R)ELGILYGCK(G)	P84809	82.0
B8, B9, B10, B11, B12	Neurotoxin Bot2 OS = Buthus occitanus tunetanus	7354.4	(−)GRDAYIAQPENCVYECAK(N)	P01483	100.0
B3, B4, B5, B6, B7, B8, B9, B13	Potassium channel toxin alpha-KTx 9.5 OS = Buthus occitanus tunetanus	2949	(K)GKHAVPTCD DGVCN(C)	P84744	85.7
B6, B7, B8, B9, B10, B11, B12, B13, B14	Potassium channel toxin BuTXK-beta OS = Buthus occitanus israelis	10,192.2	(K)YAVPESTLR(T)	B8XH40	89.8
B8	Toxin Boma6a OS = Buthus occitanus mardochei	7477.5	(R)DAYCNDLCTK(N)	P60255	85.6
**Proteins Associated with other Species**					
**Sample/Fraction**	**Protein Name**	**MW**	**Sequence**	**Swiss-Prot ID**	**% Coverage**
B8, B9, B10, B11	Alpha-insect toxin Lqq3 OS = Leirus quinquestriatus quinquestriatus	7240.2	(K)YGNACWCYALPDNVPIRVPGKCH(-)	P01487	97.7
B10	Alpha-like toxin BmK-M1 OS = Mesobuthus martensii	3598.4	(-)MCIPCFTTNPNMAAK(C)	P45697	81.0
B11, B12	Beta-insect excitatory toxin BmK IT-AP OS = Mesobuthus martensii	10,225.1	(K)VYYADK(G)	O77091	80.8
B11	Beta-insect excitatory toxin LqhITIa OS = Leirus quinquestriatus hebraeus	9900.8	(K)YCDFTIIN(-)	P68721	81.2
B12	Beta-insect excitatory toxin LqhITIb OS = Leirus quinquestriatus hebraeus	9958.8	(K)KYCDFTIIN(-)	P68722	85.4
B10, B11, B12	Beta-insect excitatory toxin LqhITIc OS = Leirus quinquestriatus hebraeus	9935.8	(K)VMEISDTR(K)	P68723	73.7
B11, B12, B13	Beta-insect excitatory toxin LqhITId OS = Leirus quinquestriatus hebraeus	9992.8	(K)VYYAEK(G)	P68724	71.7
B10, B11, B12	Beta-toxin Isom1 OS = Isometrus vittatus	7908.1	(R)KKYCDYTIIN(-)	P0C5H1	86.2
B9, B10, B11, B12	Chlorotoxin OS = Leirus quinquestriatus quinquestriatus	4004.8	(-)MCMPCFTTDHQMAR(K)	P45639	85.8
B8, B9, B10	Chlorotoxin-like peptide OS = Androctonus australis	3598.4	(-)MCIPCFTTNPNMAAK(C)	P86436	77.8
B1	L-amino-acid oxidase (Fragment) OS= Daboia russelli siamensis	46,371.9	(R)IFFAGEYTANAHGWIDSTIKSGLTAAR(D)	Q4F867	82.4
B2, B16	Neurotoxin-1″ OS = Androctonus australis	9060.8	(K)DLPDNVPIK(D)	PO1479	84.8
B13, B18	Lipolysis-activating peptide 1-alpha chain OS = Mesobuthus martensii GN = LVP1a	11,329.2	(K)YYCTILGENEYCR(K)	Q6WJF5	86.8
B14	Neurotoxin MeuNaTx-6 OS = Buthus eupeus	7995	(K)PHNCVYECFDAFSSYCNGV(C)	E7CZY9	89.4
B17	Toxin AaHIT4 OS = Androctonus australis	7786.0	(K)LACYCQGAR(K)	P21150	96.1
B19	Beta-insect excitatory toxin LqhIT1d OS = Leiurus quinquestriatus hebraeus	9992.8	(K)VYYAEK(G)	P68724	71.7
B20, B21	Beta-toxin BmKAs1 OS = Mesobuthus martensii	9802.7	(K)LACYCEGAPK(S)	Q9UAC8	87.3
B22	Beta-insect excitatory toxin LqhIT1c OS = Leiurus quinquestriatus hebraeus	9935.8	(K)VMEISDTR(K)	P68723	73.7
B12, B13	Neurotoxin-1 OS = Androctonus australis	9060.8	(K)DLPDNVPIK(D)	P01479	84.8
B8	Potassium channel toxin alpha-KTx 15.2 OS = Mesobuthus martensii	6206.6	(K)AIGVAAGK(C)	Q8I0L5	87.0
B14, B15	Potassium channel toxin alpha-KTx 3.4 OS = Leirus quinquestriatus hebraeus	4020.9	(-)GVPINVK(C)	P46110	95.2
B3, B4, B5, B6, B8, B9, B13	Potassium channel toxin alpha-KTx 8.5 OS = Odontobuthus doriae	3188.6	(-)VSCEDCPEHCSTQK(A)	P0CC12	82.2
B8, B9	Potassium channel toxin BmTXK-beta OS = Mesobuthus martensii	10,430.6	(K)AIGKCEDTECK(C)	Q9NJC6	90.6
B11	Potassium channel toxin MeuTXKbeta3 OS = Buthus eupeus	10,338.2	(K)YAVPESTLR(T)	A9XE60	86.1
B12, B13	Toxin AaHIT4 OS = Androctonus australis	7786.0	(K)LACYCQGAR(K)	P21150	96.1
B10, B11	Toxin Aam2 OS = Androctonus amoreuxi	9283	(K)NGAESGYCQWFGRYGNA(C)	Q86SE0	77.9
B8	Toxin BmKa3 OS = Mesobuthus martensii	9425.9	(K)LPDKVPIR(V)	Q9GUA7	85.7
B13	Toxin Isom2 OS = Isometrus vittatus	7884.75	(K)VHYADKGYCCLLSCY(C)	P0C5H2	98.7
B8, B9	Toxin Lqh4 OS = Leirus quinquestriatus hebraeus	7220.3	(K)YGNACWCIK(L)	P83644	89.6

## Data Availability

Not applicable.
